# A physical optics formulation of Bloch waves and its application to 4D STEM, 3D ED and inelastic scattering simulations

**DOI:** 10.1107/S2053273325000142

**Published:** 2025-01-30

**Authors:** Budhika G. Mendis

**Affiliations:** a Department of Physics Durham University South Road Durham DH1 3LE United Kingdom; Helmholtz Centre for Infection Research, Germany

**Keywords:** Bloch waves, multislice, 4D STEM simulation, precession electron diffraction, phonon scattering, plasmon scattering, inelastic scattering

## Abstract

Bloch waves are often used in dynamical diffraction calculations, such as simulating electron diffraction intensities for crystal structure refinement. However, this approach relies on matrix diagonalization and is therefore computationally expensive for large unit cell crystals. Here Bloch wave theory is re-formulated using the physical optics concepts underpinning the multislice method. In particular, the multislice phase grating and propagator functions are expressed in matrix form using elements of the Bloch wave structure matrix. The specimen is divided into thin slices, and the evolution of the electron wavefunction through the specimen calculated using the Bloch phase grating and propagator matrices. By decoupling specimen scattering from free space propagation of the electron beam, many computationally demanding simulations, such as 4D STEM imaging modes, 3D ED precession and rotation electron diffraction, phonon and plasmon inelastic scattering, are considerably simplified. The computational cost scales as  per slice, compared with  for a standard Bloch wave calculation, where *N* is the number of diffracted beams. For perfect crystals the performance can at times be better than multislice, since only the important Bragg reflections in the otherwise sparse diffraction plane are calculated. The physical optics formulation of Bloch waves is therefore an important step towards more routine dynamical diffraction simulation of large data sets.

## Abstract 2

This article describes the re-formulation of Bloch waves using physical optics theory for more efficient simulation of large electron diffraction data sets.

## 1. Introduction

Data-intensive electron microscopy methods, such as 4D scanning transmission electron microscopy (4D STEM) and 3D electron diffraction (3D ED), require large-scale computing of dynamical diffracted intensities for quantitative analysis (Ophus, 2019 ▸; Gemmi *et al.*, 2019 ▸). Adding inelastic scattering events, in particular phonons (Forbes *et al.*, 2010 ▸; Zeiger & Rusz, 2020 ▸) and plasmons (Mendis, 2019 ▸; Barthel *et al.*, 2020 ▸), increases the computational complexity even further. The multislice algorithm (Cowley & Moodie, 1957 ▸; Kirkland, 2010 ▸) is often used for dynamical diffraction calculations, especially if the sample is thin and non-periodic. However, multislice is impractical for (say) 3D ED tomography simulations, so that Bloch waves must be used instead (Palatinus *et al.*, 2015 ▸; Klar *et al.*, 2023 ▸). There have been several attempts to increase the speed of dynamical diffraction calculations. For example, Ophus (2017 ▸) introduced the PRISM algorithm for 4D STEM simulations. Instead of simulating every STEM probe position, multislice simulations are performed for a limited number of partial plane waves within the STEM probe, which are then used to construct the exit wave at each scan position. Similarly, Mendis (2024*a* ▸) proposed a scattering cluster algorithm, that replaced the computationally expensive eigen-decomposition routine in Bloch waves to a simpler matrix multiplication operation. For inelastic scattering, the mixed static potential method of Peters (2021 ▸) reduces the number of phase grating calculations required for a frozen phonon multislice simulation. Finally, Mendis (2023 ▸) introduced a phase scrambling algorithm (PSA), which modelled all inelastic scattering in a multislice simulation simultaneously, with a random phase introduced to preserve incoherence between inelastic events.

Both multislice and Bloch wave theories are derived from the Schrödinger equation (Kirkland, 2010 ▸) and are therefore equivalent. In this paper, the close relationship between the two theories is examined in more detail. In particular, the multislice phase grating and propagator functions in reciprocal space are expressed as matrices, containing off-diagonal and diagonal Bloch wave structure matrix elements, respectively. The decoupling of specimen transmission and free space electron propagation enables efficient Bloch wave simulation of computationally demanding applications, such as 4D STEM, 3D ED and inelastic scattering. A common feature in all these examples is that the specimen remains fixed, while only the electron wavevector undergoes any change. For example, PRISM simulates individual partial plane waves within the STEM probe for an identical specimen, while for phonon and plasmon inelastic scattering, the diffuse background intensity is modelled by a change in the incident electron wavevector (Mendis, 2024*b* ▸). A physical optics approach to Bloch wave simulations is therefore proposed, where matrices, consisting of Bloch wave structure matrix elements, replace the phase grating and propagator functions in multislice. The Bloch phase grating matrix must be calculated only once for the (fixed) specimen. The specimen is divided into a series of thin slices, and the evolution of the incident electron wavefunction within the specimen calculated by applying the Bloch phase grating and propagator matrices for each slice. The computational complexity of a physical optics Bloch wave simulation therefore scales in a similar manner to multislice. Furthermore, computational techniques such as PRISM and PSA, which were originally developed for multislice, can also be extended to Bloch wave theory via this method.

The organization of the paper is as follows. In Section 2 the physical optics theory of Bloch wave scattering is presented. Applications to 3D ED, 4D STEM and phonon, plasmon inelastic scattering calculations are also discussed. Section 3 covers simulation details, while results are presented in Section 4. The accuracy of physical optics Bloch wave simulations is compared with that of multislice, the two theories being mathematically equivalent, although numerical differences remain due to practical limitations in the implementation of each technique. A summary is provided in Section 5.

## 2. Mathematical background

### 2.1. Physical optics description of Bloch wave scattering

In Bloch wave theory the electron wavefunction at specimen depths *z* and 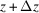 are related by (Hirsch *et al.*, 1965 ▸; Spence & Zuo, 1992 ▸)
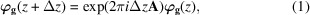
where 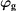 is a column vector comprising the Fourier transform coefficients of the electron wavefunction. Bragg beam intensities are expressed as the square modulus 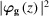. 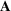 is the so-called structure matrix, defined by
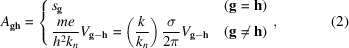
where 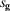 is the deviation parameter for the Bragg beam 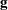 and *k* is the electron wavenumber within the specimen, with component 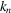 along the specimen surface normal direction. Specimen tilting changes all elements in the structure matrix; the diagonal terms via 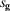 and non-diagonal terms via 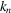. 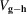 is the Fourier coefficient of the crystal potential. Note that the mean inner potential 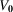 does not appear in the structure matrix, since this is included as a (small) correction to the electron wavenumber *k* (Hirsch *et al.*, 1965 ▸; Spence & Zuo, 1992 ▸). The mean inner potential represents a uniform background and therefore for the present discussion it is convenient to set 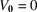, since the Bragg beam intensities do not depend on the precise value of 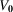. The other physical constants are Planck’s constant *h*, mass (*m*) and charge (*e*) of an electron. The interaction constant is defined by σ = 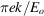, where 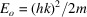 is the incident electron kinetic energy.

Equation (1) is usually solved by diagonalizing the structure matrix, *i.e.*
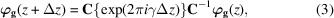
where 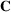 is a square matrix of all eigenvectors. Since 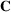 is unitary, its inverse 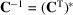, where T denotes matrix transpose and the asterisk sign the complex conjugate (Hirsch *et al.*, 1965 ▸). 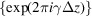 is a diagonal matrix, with γ being the eigenvalues of 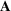. Eigen-decomposition is computationally expensive, with the complexity scaling as 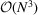 for an 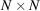 square matrix. Note that computational complexity is defined here as the number of arithmetic operations required to calculate a given quantity directly. The actual algorithm used for calculations may however have fewer arithmetic operations.

Let us now assume normal plane wave incidence, *i.e.*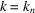, and express the structure matrix as the sum of two simpler matrices:
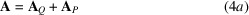

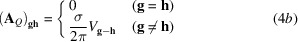

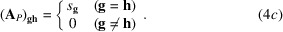


The hollow matrix 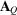 consists of only the non-diagonal terms of the structure matrix, which contain specimen-related information. In contrast, the diagonal matrix 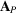 consists of only the diagonal elements of 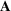 that describe electron beam propagation via the deviation parameter. Provided Δ*z* is small equation (1) approximates to
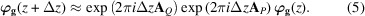
The approximation is because 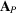 and 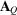 are non-commuting matrices. The above equation should be compared with the equivalent multislice result for normal plane wave incidence (Kirkland, 2010 ▸):
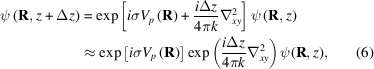
where 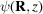 is the real-space electron wavefunction at specimen depth *z* and position vector 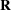 in the plane perpendicular to the electron optic axis. 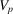 is the projected potential and 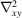 = 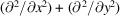 is the Laplacian operator. The approximation is due to the fact that the 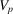 and 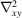 operators do not commute. Equation (6) must be Fourier transformed for direct comparison with equation (5). It can be shown that (Kirkland, 2010 ▸)
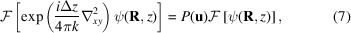
where *P* is the propagator function defined by
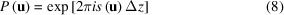
with 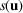 being the deviation parameter at the reciprocal vector 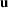. Comparing equations (7), (8) with equations (4*c*) and (5), and noting that at normal plane wave incidence the Fourier transform of 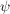 has coefficients 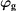, it is clear that
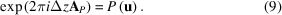
In other words, 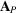 is directly related to the multislice propagator function in reciprocal space. For equation (5) to be consistent with equation (6) we must therefore have
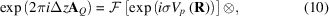
where 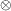 denotes the convolution operation. 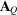 therefore determines the phase grating function in multislice. A proof of equation (10) will now be presented. The projected potential is
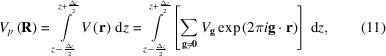
where 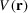 is the crystal potential at position vector 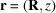, which can be expressed as a Fourier series due to the crystal periodicity. Note that the mean inner potential is omitted, to be consistent with the Bloch wave result 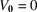. Writing 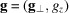, where 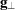 and 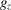 are, respectively, components of 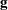 normal and parallel to the electron optic axis, we obtain
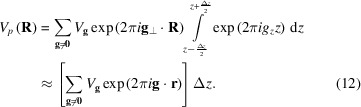


The approximation in equation (12) is valid in the limit of small Δ*z*. Therefore
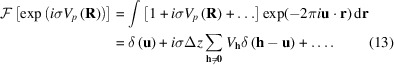
In the last line of equation (13) a dummy reciprocal vector 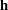 is used to replace 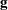, for reasons that will become clear later on. δ symbols represent Dirac delta functions. The result of convolving equation (13) with a function 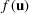 that has the same periodicity in reciprocal space as the crystal is given by
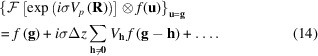



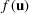
 may, for example, represent the right-hand side of equation (7); equation (14) would then be the Fourier transform of equation (6). Making the transformation 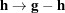:
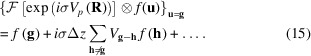


Consider now the left-hand side of equation (10). Writing the periodic function 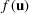 as a column vector over reciprocal vectors 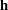 we have
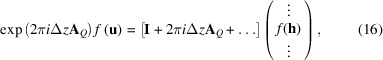
where 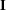 is the identity matrix. From equation (4*b*[Disp-formula fd4b]) it is clear that the first two terms in equations (15) and (16) are equal. Similar arguments can be used to demonstrate that the higher-order terms are equal as well (see the supporting information). This completes the proof of equation (10).

Before concluding this section, it is instructive to examine the error involved in using a physical optics description of Bloch wave scattering. We have
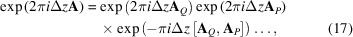
where [ ] is the commutator bracket. It is easy to show that
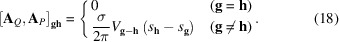
The commutator matrix contains both specimen and beam propagation information coupled, and therefore lacks a straightforward physical interpretation. Chen & Van Dyck (1997 ▸) have proposed more accurate multislice methods, where a similar mixed operator is used to model electron beam propagation within the specimen potential, as opposed to free space propagation. Higher-order terms in equation (17) will introduce further, more complicated, corrections. In Sections 2.2 to 2.4, application of the physical optics Bloch wave method to different simulations, namely 3D ED, 4D STEM and inelastic scattering, will be described in more detail.

### 2.2. 3D ED and computational complexity

Precession electron diffraction (PED) and continuous rotation electron diffraction (cRED) are often used to suppress dynamical diffraction in 3D ED measurements (Vincent & Midgley, 1994 ▸; Nederlof *et al.*, 2013 ▸). In PED, the electron beam is precessed in a hollow cone at fixed specimen orientation. In a standard quantum-mechanical Bloch wave simulation equation (1) must be solved for every incident wavevector, and the Bragg diffracted intensities incoherently summed to give the final result. For *N* diffracted beams the computational cost for diagonalizing the structure matrix 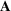 is 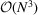 for every incident wavevector. Compare this with the physical optics approach given by equation (5). Since 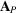 is a diagonal matrix [equation (4*c*[Disp-formula fd4c])]:
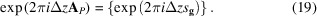


The right-hand matrix consists only of 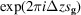 terms along the diagonal and zero elsewhere. Since 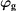 is an 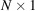 column vector, propagation of electron beams between slices, which is given by 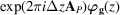 in equation (5), requires only *N* multiplications. On the other hand, multiplication by the Bloch phase grating matrix 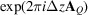 requires 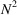 multiplications; the greater complexity for this step is because of the convolution operation in equation (10). In practice, 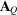 has to be diagonalized first to evaluate 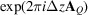, although since the specimen is fixed this can be re-used in the simulation. If there are a relatively small number of beams (*e.g.* Si [001] in this work; Section 4.1) then 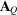 is diagonalized only once. However, in PED of large unit cell crystals the total number of Bragg beams can be quite large, and therefore it is desirable to select only a subset of strongly excited beams for any given incident wavevector. A single diagonalization of 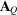 would then only be applicable for a limited wavevector range. Outside this range, 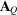 would have to be diagonalized again for the new set of Bragg beams.

Assuming 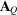 diagonalization is a one-off calculation, the computational cost for physical optics Bloch waves is 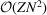 per incident PED wavevector, where *Z* is the number of slices into which the specimen is divided. The physical optics implementation of Bloch wave scattering is therefore more efficient than a quantum-mechanical calculation provided 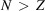, which is satisfied for thin specimens and large unit cell crystals. For perfect crystals, the performance can also at times be more efficient than multislice, which scales as 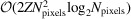 due to the fast Fourier transform algorithm applied to square images of size 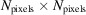. The improvement is because only the important Bragg reflections are calculated with Bloch waves, while multislice simulates the entire diffraction plane, which is inherently sparse for a perfect crystal.

In cRED the diffraction patterns for a fixed incident electron wavevector at multiple specimen tilts are incoherently summed. Specimen tilting alters both 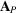 and 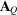 matrices. However, for the latter the change is simply a multiplicative constant 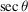, since 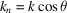 [equation (2)]. The new eigenvalues of 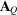 change by the same factor, while the eigenvectors remain unchanged. Therefore, earlier comments on re-using the 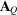 diagonalization apply to cRED as well, *i.e.* diagonalization is performed only once for specimen tilts that share the same set of Bragg beams. The computational complexity of cRED is hence similar to that of PED.

### 2.3. 4D STEM simulation procedure

The PRISM algorithm (Ophus, 2017 ▸) for fast simulation of 4D STEM data sets can be implemented using the physical optics Bloch wave method. The electron wavefunction for a STEM probe incident at position 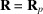 is given by (Mendis, 2015 ▸)
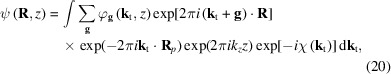
where 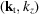 are the transverse and longitudinal wavevector components of the STEM probe partial plane waves and χ is the lens aberration function. 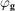 is the diffracted beam wavefunction for a given partial plane wave, *i.e.* equation (1) or (5). The integration is carried out over the STEM probe aperture. The diffraction pattern is obtained by Fourier transforming equation (20) at the specimen exit plane *z* = *t*, where *t* is the specimen thickness:
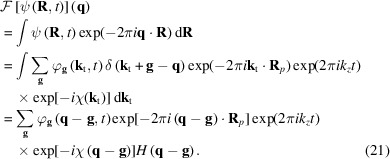

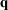
 is a 2D reciprocal vector in the diffraction plane. 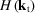 is a STEM probe aperture function, which is equal to unity for any partial plane wave 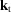 within the aperture and zero outside it. For most STEM measurements it is reasonable to assume that 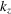 is approximately constant, so that equation (21) simplifies to
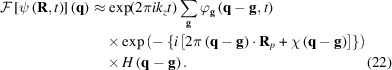


Equation (22) suggests an efficient route for 4D STEM physical optics Bloch wave simulations. For a given STEM probe partial plane wave 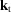 the diffracted beams at the specimen exit surface are calculated by applying equation (5) iteratively for individual slices. From this the diffraction pattern at STEM probe position 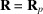 readily follows, *i.e.* for every diffracted beam 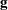, 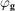 at the exit surface is multiplied by the phase 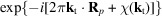 and assigned to the reciprocal vector 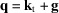. The process is repeated for every partial plane wave within the STEM probe aperture. For overlapping discs, more than one combination of 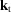 and 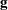 can contribute to 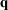 in the overlapped region. Their coherent summation according to equation (22) gives rise to interference effects. The intensity of the final diffraction pattern is obtained by taking the square modulus. The ‘constant’ phase term 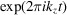 can be ignored, since it drops out of the intensity. Note that the STEM probe position 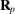 only appears as a phase term 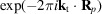, so that the diffraction patterns for all probe positions can be simulated in parallel. Furthermore, the Bloch phase grating matrix needs also to be calculated only once, which further reduces the computing time.

### 2.4. Monte Carlo simulation of inelastic scattering

The Monte Carlo method, as applied to inelastic scattering, has been discussed in a number of previous publications (Mendis, 2019 ▸, 2024*b* ▸). Here the procedure for simulating the diffuse scattered intensity background due to single phonon or plasmon scattering is summarized. The first step is to divide the sample into a series of slices, which represent the depths at which inelastic scattering events occur. Since the phonon and plasmon inelastic mean free paths are relatively long (Vos & Winkelmann, 2019 ▸), the slice thickness can be made larger than multislice or physical optics Bloch wave calculations, without any significant loss of accuracy. The incident electron beam is elastically propagated to a given inelastic scattering depth *s*, using either equation (1) or (5). Following inelastic scattering, there will be a change in the direction of the incident electron wavevector, characterized by the polar (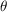) and azimuthal (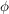) scattering angles. The wavenumber of the high-energy electron is assumed to be constant for low energy loss phonon and plasmon excitations. Furthermore, for low-angle scattering the ratio 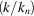 in equation (2) is approximately equal to unity. Therefore, only the matrix 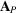 is updated following inelastic scattering. The inelastic scattered electron is elastically propagated (using the new Bloch propagator matrix) to the specimen exit surface (depth *t*), and the diffracted beam 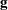 assigned to the reciprocal vector 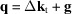 in the diffraction plane, where 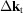 is the change in the transverse wavevector component of the electron due to inelastic scattering. Incoherently summing all inelastic scattering events from all depths gives the final diffraction pattern intensity *I*:
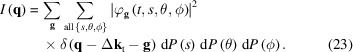
The Dirac delta term ensures momentum or wavevector conservation following inelastic scattering. 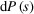 is the probability of inelastic scattering between depths *s* and 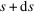, and similarly for 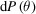 and 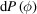. They can be estimated from the relevant differential scattering cross section (σ). For uncorrelated phonons this would be the thermal diffuse scattering (TDS) from a single atom (Pennycook & Jesson, 1991 ▸):
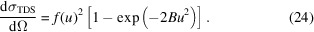

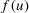
 is the atom scattering factor at scattering vector magnitude *u*, *B* is the Debye–Waller factor and 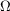 is the scattering solid angle. It can be shown that for uncorrelated phonons (Mendis 2024*b* ▸)
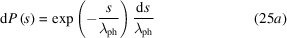

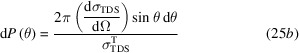

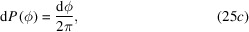
where 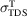 is the total TDS cross section, obtained by integrating equation (24) over all scattering angles. For a mono-atomic solid the phonon mean free path 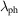 is 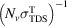, with 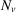 being the atomic number density (Mendis, 2024*b* ▸). A differential scattering cross section for plasmons was proposed by Ferrell (1956 ▸) based on a harmonic oscillator model:
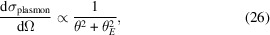
where 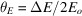 is the characteristic scattering angle for plasmon energy loss 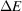 (Egerton, 1996 ▸). Due to the small value of 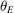 (*e.g.* 0.04 mrad for Si at 200 kV electron beam voltage) the plasmon diffuse scattering is confined to small scattering angles. Furthermore, for plasmon scattering (Mendis, 2024*b* ▸)
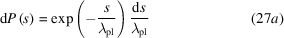

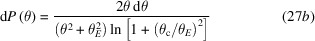

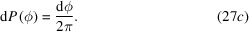
The plasmon mean free path 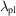 can be measured using electron energy loss spectroscopy (Mendis, 2019 ▸). 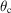 is the critical scattering angle above which plasmon excitation is damped (Egerton, 1996 ▸); it too can be experimentally measured using energy filtered diffraction (Bertoni *et al.*, 2011 ▸).

Equation (23) is very time-consuming to calculate due to the large number of possible inelastic scattering configurations 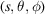. This is especially true for the TDS background, which can have large momentum transfer. Since the same Bloch phase grating matrix is used throughout, a physical optics Bloch wave simulation is more efficient than a standard quantum-mechanical calculation, where a new structure matrix has to be diagonalized after every inelastic scattering event (Mendis, 2024*b* ▸).

## 3. Simulation methods

All simulations were performed on a 500 Å-thick, Si [001] specimen at 200 kV incident electron beam voltage. A total of 1681 zero-order Laue zone (ZOLZ) Fourier coefficients were used for Bloch wave simulations, with the crystal potential calculated using Kirkland’s (2010 ▸) atom scattering factors. In all cases Bloch wave results were compared against multislice simulations. The size of the (square) multislice supercell was 10*a*_o_, with a slice thickness of *a*_o_/4, where *a*_o_ is the Si unit-cell parameter. Kirkland’s (2010 ▸) atom scattering factors were used to calculate the projected potential, sampled over 1024 × 1024 pixels.

For PED the beam precession angle was 2° and 500 incident wavevectors with uniformly spaced azimuthal angles were simulated. Tilted incident wavevectors do not satisfy periodic boundary conditions, leading to potential aliasing artefacts in multislice. The real-space electron wavefunction was therefore multiplied by a Hanning window, prior to calculating the diffraction pattern. For 4D STEM simulations a 15 mrad probe semi-convergence angle was assumed with all electron-optic aberrations set to zero. In Bloch wave calculations the STEM probe was approximated by a total of 13234 partial plane waves, while for multislice the STEM probe wavefunction (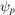) at the specimen entrance surface was calculated using the following semi-analytical equation (Mendis, 2015 ▸):
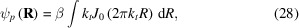
where 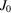 is the zero-order Bessel function of the first kind and β is a normalization constant for the probe intensity. The probe wavefunction converges faster for equation (28) compared with the partial plane wave method.

For inelastic scattering simulations the calculated (uncorrelated) phonon mean free path 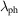 was 7724 Å. 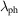 is inversely proportional to the total TDS scattering cross section 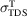. Kirkland’s (2010 ▸) atom scattering factors are however only strictly valid up to 12 Å^−1^. Nevertheless, this corresponds to a sufficiently large scattering angle of 301 mrad at 200 kV, so that the scattering vector can be truncated without significantly affecting the value of 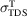 or 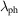. The plasmon mean free path of 1050 Å was measured by experiment (Mendis, 2019 ▸). 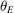 was calculated to be 0.04 mrad for a 17 eV plasmon energy loss in silicon. By comparing experiment with simulation, Barthel *et al.* (2020 ▸) estimated a 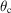 value of 15.0 mrad at 300 kV, which is equivalent to 19.1 mrad at 200 kV.

The 500 Å-thick sample was divided into ten slices and it was assumed that inelastic scattering occurred in the middle of each slice. The single scatter diffuse intensity was then calculated using the physical optics Bloch wave model, as described in Section 2.4. The equivalent calculation in multislice proceeds as follows. The change in beam direction following inelastic scattering effectively multiplies the real-space electron wavefunction by a phase ramp term 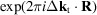 (Barthel *et al.*, 2020 ▸). To minimize aliasing artefacts 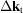 is rounded to the nearest pixel in the diffraction plane. The electron wavefunction must also be multiplied by the probabilities 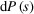, 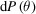 and 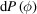, *i.e.* equations (25*a*)–(25*c*) for phonons and equations (27*a*)–(27*c*) for plasmons. The reciprocal-space pixel size (d*q*) determines the resolution 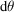 and 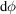 in polar and azimuthal scattering angles. For example, 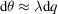 and 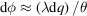, where λ is the electron wavelength (Mendis, 2023 ▸). The inelastic electron wavefunction is then elastically propagated to the specimen exit surface. Incoherently summing the multislice diffraction patterns for all inelastic scattering events gives the single scatter diffuse background.

The quantum excitation of phonons model (Forbes *et al.*, 2010 ▸) was also used to simulate TDS, assuming frozen phonons consisting of independently vibrating atoms. The electron beam was quasi-elastically propagated through a silicon supercell where the atoms were randomly shifted from their equilibrium lattice positions. The atom displacements followed a Gaussian distribution with root mean square displacement of 0.078 Å (Kirkland, 2010 ▸). A total of 100 frozen phonon configurations were simulated to obtain statistically valid results. The phonon TDS is obtained by subtracting the coherently summed diffraction pattern from the incoherent diffraction pattern (Forbes *et al.*, 2010 ▸).

## 4. Results and discussion

### 4.1. Precession electron diffraction (PED)

**Figure 1:**
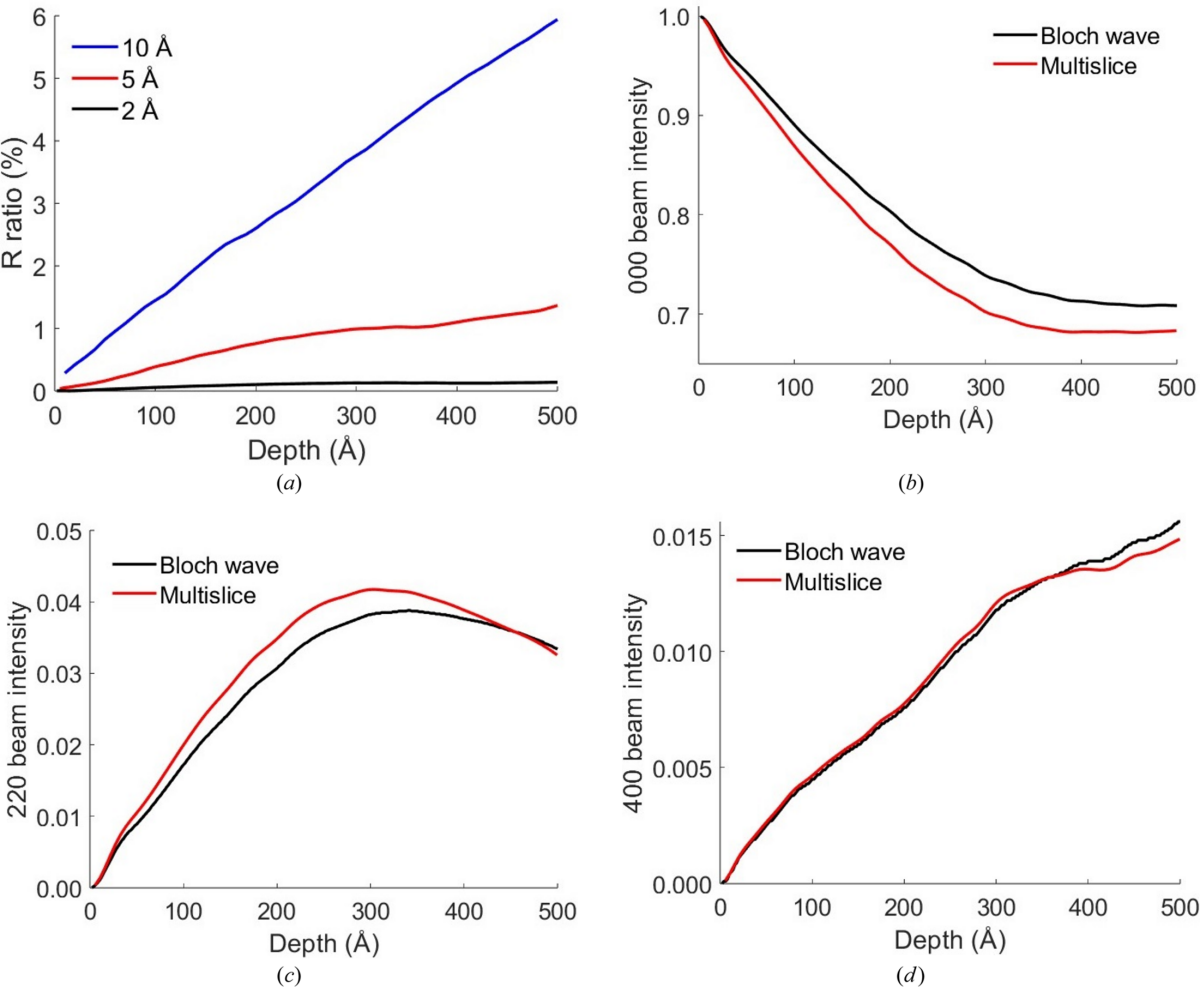
PED simulation results for a 500 Å-thick Si [001] specimen with 2° beam precession angle. (*a*) *R* ratio [equation (29)] for diffracted beam intensities plotted as a function of specimen depth. The Bragg beam intensities were calculated using the physical optics Bloch wave method for slice thicknesses of 10, 5 and 2 Å. A total of 441 reflections, including those kinematically forbidden by the diamond glide plane, were used to calculate the *R* ratio. Comparison of the (*b*) 000, (*c*) 220 and (*d*) 400 reflection intensity *pendellösung* calculated using physical optics Bloch wave (2 Å slice thickness) and multislice.

PED is used to test convergence of the physical optics Bloch wave model [equation (5)] with respect to slice thicknesses (*i.e.* 10, 5 and 2 Å) by comparing with the (more accurate) quantum-mechanical result [equation (1)]. Bragg beam intensities are calculated at each specimen depth and convergence is measured using the formula
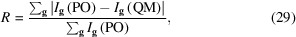
where 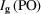, 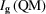 are the Bloch wave physical optics and quantum-mechanical diffracted beam intensities, respectively. The ‘| |’ symbol denotes the absolute value. All *hkl* reflections apart from those forbidden by lattice centring (*i.e. hkl* mixture of even and odd indices) were used to calculate the *R* ratio (441 total number of reflections). The excluded reflections do not appear even under dynamical diffraction. Reflections kinematically forbidden by the diamond glide plane in silicon, such as 200, can appear dynamically under suitable conditions, and were therefore included in the *R* ratio. Fig. 1 ▸(*a*) shows the *R* ratio plotted as a function of specimen depth for the different slice thicknesses. For a 2 Å slice thickness the *R* ratio is less than 0.14% at all depths, *i.e.* significantly better than current experimental crystal structure refinements using electron diffraction (Palatinus *et al.*, 2015 ▸; Klar *et al.*, 2023 ▸). The 2 Å slice thickness is therefore considered to give converged results and is used throughout the rest of this paper.

Because of their close similarities, it is of interest to compare the (converged) physical optics Bloch wave results with multislice. Fig. 1 ▸(*b*) shows the PED intensity *pendellösung* for the unscattered 000 beam, while Figs. 1 ▸(*c*) and 1 ▸(*d*) are the equivalent plots for two example Bragg diffracted beams, namely the 220 and 400 reflections, respectively. The overall shape of the *pendellösung* is similar for both methods, although there are also clear numerical differences. There are potentially two sources of error in the multislice simulations, both related to the large tilt angle (2°) of the precessed beam. The first is that the incident wavevectors do not satisfy periodic boundary conditions. However, doubling the supercell size did not have any significant effect on the multislice intensities, suggesting that the error due to non-periodic boundary conditions is small. Secondly, the standard multislice propagator function (Kirkland, 2010 ▸) is strictly valid for normal beam incidence. Chen & Van Dyck (1997 ▸) have proposed advanced multislice schemes for tilted beams, although this is outside the scope of the present work and was not investigated. As for Bloch waves, errors can be introduced due to neglecting HOLZ (higher-order Laue zone) reflections in the calculation. This is however not a fundamental limitation of the Bloch wave method; HOLZ reflections were not included since for zone-axis orientations they typically have only a small effect on the accuracy (Spence & Zuo, 1992 ▸), while the computing time increases significantly.

### 4.2. 4D STEM

**Figure 2:**
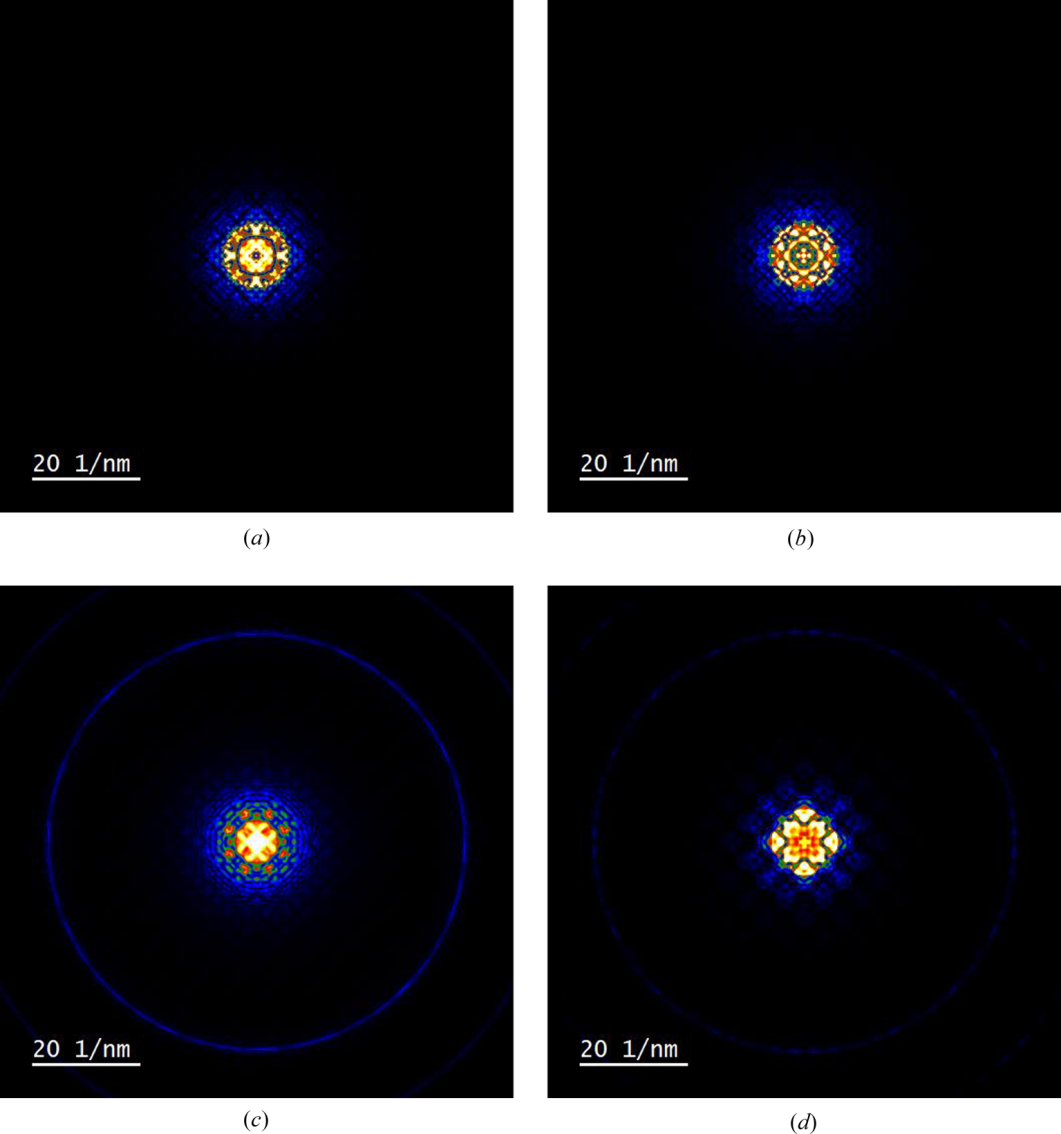
Physical optics Bloch wave simulated electron diffraction patterns for a 15 mrad semi-convergence angle, aberration-free STEM probe positioned (*a*) on an atom column and (*b*) off an atom column in a 500 Å-thick Si [001] specimen. The two STEM probe positions are indicated by the crosses labelled ‘A’ and ‘B’ in Fig. 3 ▸(*a*). The corresponding results from a multislice simulation are shown in (*c*) and (*d*), respectively. All diffraction patterns are displayed on a square root intensity scale to highlight weak features.

**Figure 3:**
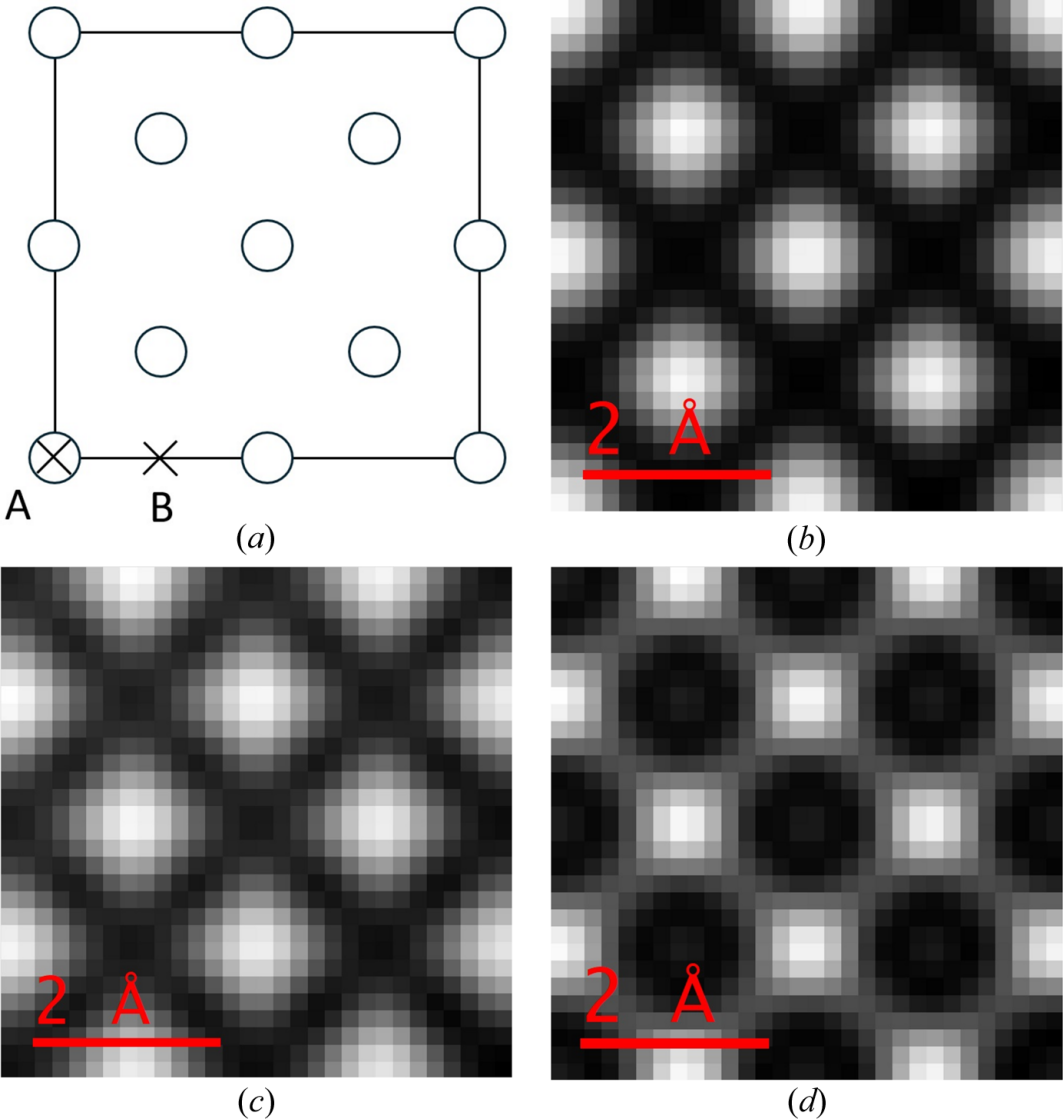
(*a*) Schematic of a single silicon unit cell in [001] projection. Open circles denote end-on atom columns. The crosses labelled ‘A’ and ‘B’ are STEM probe positions simulated in Fig. 2 ▸. Physical optics Bloch wave simulated 4D STEM images for (*b*) bright-field (BF), (*c*) annular bright-field (ABF) and (*d*) medium-angle annular dark-field (MAADF) imaging modes. The collection angles are 0–5 mrad for BF, 10–15 mrad for ABF and 30–50 mrad for MAADF. The aberration-free STEM probe semi-convergence angle was 15 mrad. The specimen is 500 Å-thick silicon, and the field of view spans a single unit cell in [001] projection.

Figs. 2 ▸(*a*) and 2 ▸(*b*) are the physical optics Bloch wave simulated diffraction patterns for a 15 mrad STEM probe positioned on and off a Si [001] atom column [positions ‘A’ and ‘B’ marked with crosses in Fig. 3 ▸(*a*), respectively]. The equivalent multislice results are shown in Figs. 2 ▸(*c*) and 2 ▸(*d*), respectively. There is considerable fine structure within the unscattered beam disc, partly due to interference with overlapping Bragg discs. The precise details of the intensity pattern are however different between the two simulation methods. One source of error is the HOLZ ring and any associated HOLZ lines within the unscattered beam disc, which are reproduced in multislice, but not in the Bloch wave simulation (Section 4.1). Finite sampling of the STEM probe partial plane waves could also potentially lead to inaccuracies. For example, the Bloch wave STEM probe wavefunction at the specimen entrance surface showed slight deviations from the perfect radial symmetry predicted by equation (28) (see the supporting information). However, separate tests confirmed that the Bloch wave diffraction patterns had converged with respect to STEM probe sampling, so that it may be ruled out as a dominant source of error.

**Figure 4:**
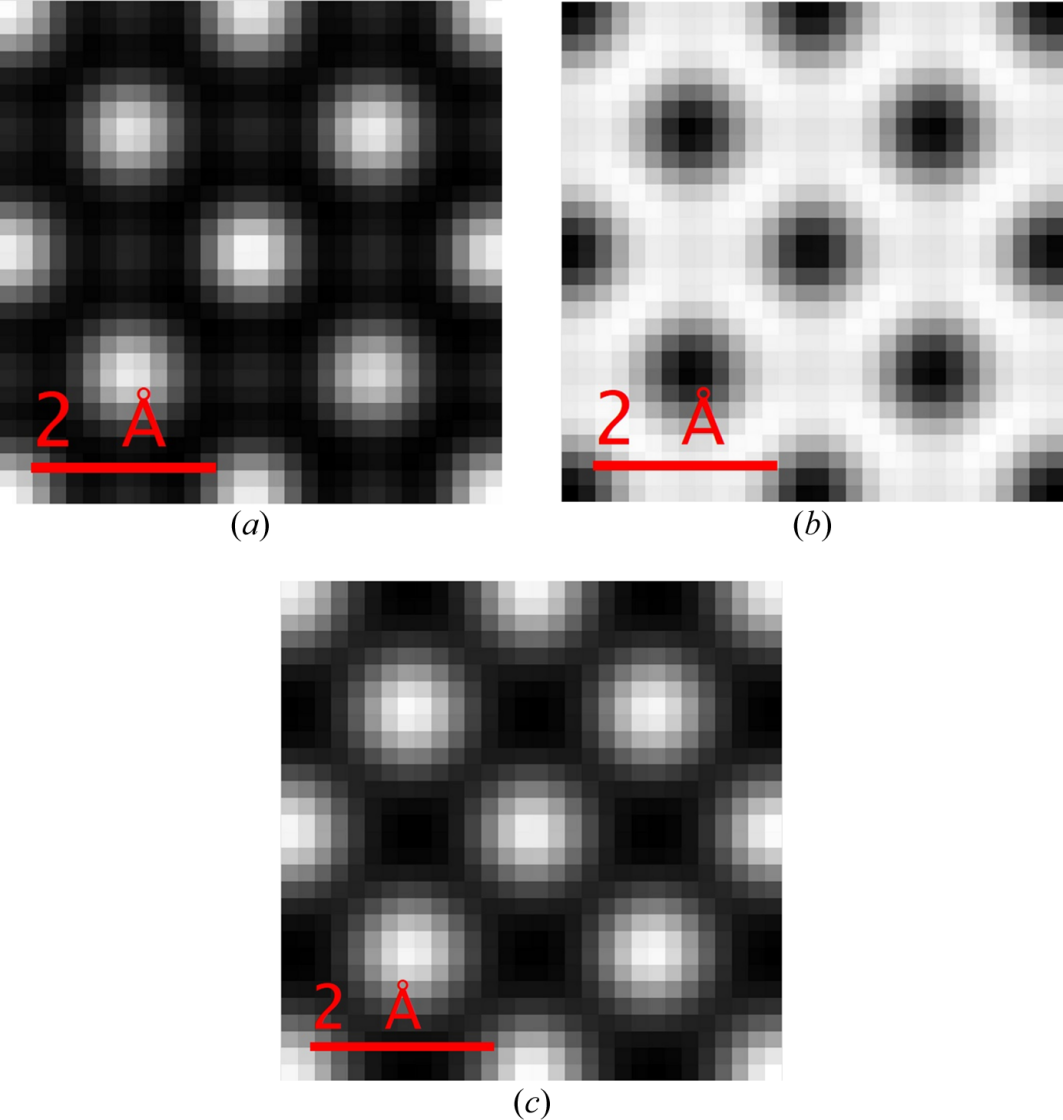
Multislice simulated 4D STEM images for (*a*) BF, (*b*) ABF and (*c*) MAADF imaging modes. The collection angles are 0–5 mrad for BF, 10–15 mrad for ABF and 30–50 mrad for MAADF. The aberration-free STEM probe semi-convergence angle was 15 mrad. The specimen is 500 Å-thick silicon, and the field of view spans a single unit cell in [001] projection.

Equation (22) and the physical optics Bloch wave method were used to calculate 4D STEM images, specifically bright-field (BF; β = 0–5 mrad), annular bright-field (ABF; β = 10–15 mrad) and medium-angle annular dark-field (MAADF; β = 30–50 mrad). β is the collection angle for each imaging mode and the simulated images are shown in Figs. 3 ▸(*b*) to 3 ▸(*d*). The results are consistent with quantum-mechanical Bloch wave calculations (see the supporting information). The images span a single silicon unit cell in [001] projection, a schematic of which is shown in Fig. 3 ▸(*a*). An important feature of equation (22) is that all probe scan positions are calculated in parallel, thereby significantly reducing the computation time. For the simulation parameters in this study the BF image displays ‘white’ atom contrast, *i.e.* intensity maxima at the atom column positions, while the contrast is inverted for the ABF and MAADF images. The equivalent multislice simulated 4D STEM images are shown in Fig. 4 ▸. The sign of BF and ABF atomic contrast is the same for both Bloch wave and multislice, although there are subtle differences in the intensity distribution. For example, there are weak subsidiary maxima between the atom columns for the multislice BF image, while the atom columns appear more rounded in the multislice ABF image compared with Bloch waves. MAADF images show more pronounced differences, such as opposite signs for the atomic column contrast. The MAADF signal is however more than an order of magnitude smaller than the BF signal, and therefore larger differences are perhaps expected, given that the Bloch wave simulations do not exactly agree with multislice (Fig. 2 ▸).

### 4.3. Plasmon and phonon inelastic scattering

**Figure 5:**
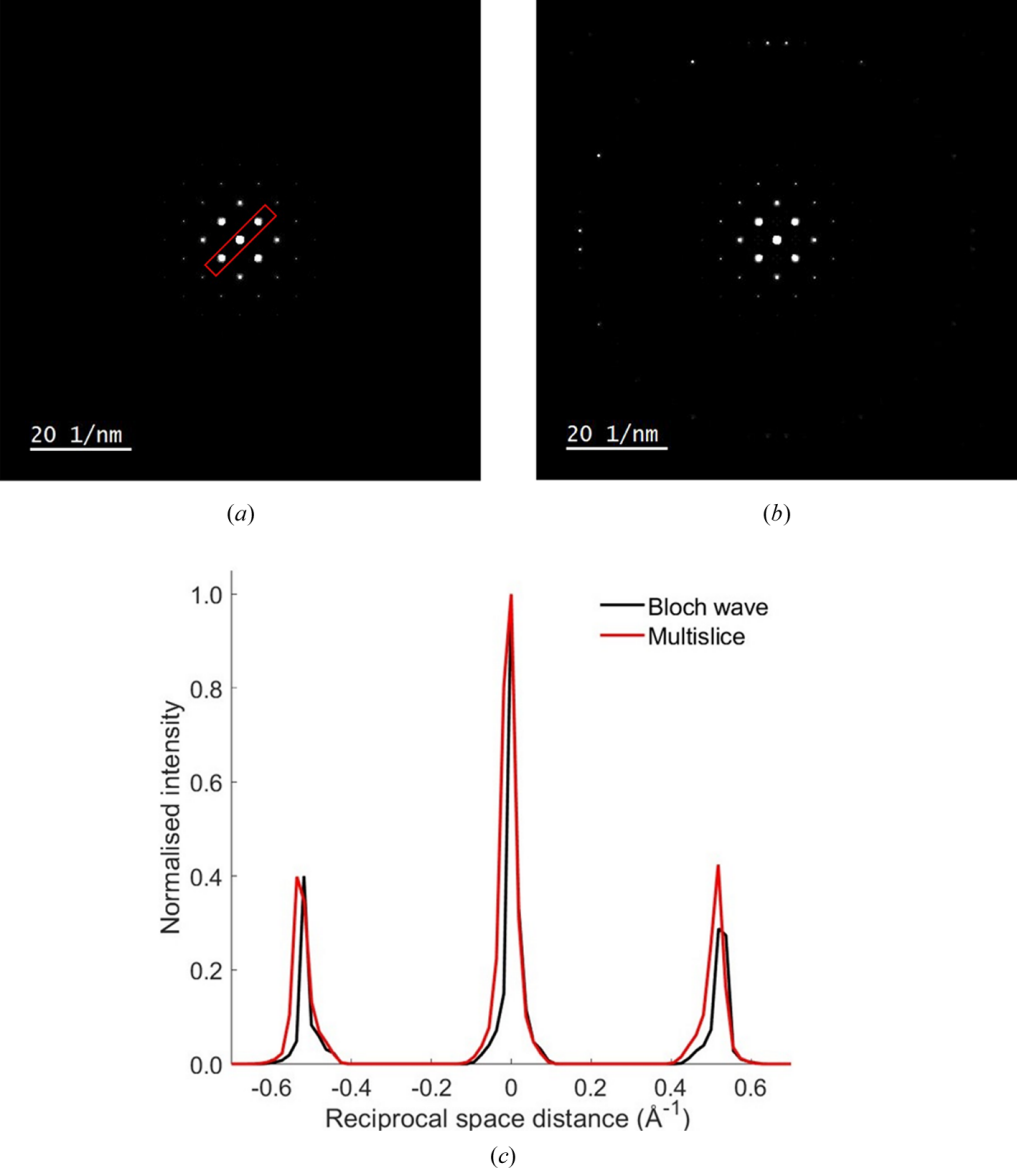
Single scatter plasmon intensity distribution for a 500 Å-thick Si [001] specimen simulated using (*a*) physical optics Bloch wave and (*b*) multislice methods. In (*c*) the intensity profile along the 220 reciprocal direction is compared for each simulation. The intensity was extracted from the annotated box region shown in (*a*). The unscattered beam is at the origin of the graph and is normalized for a direct visual comparison.

Fig. 5 ▸(*a*) is the single scatter plasmon diffuse background, calculated using the physical optics Bloch wave equation (23). The small plasmon characteristic scattering angle 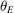 gives rise to intensity ‘halos’ around the otherwise sharp Bragg spots, which are especially visible for the innermost reflections. The broadening of the diffraction spots is consistent with experimental observations (Mendis, 2024*b* ▸). The equivalent multislice simulated result is shown in Fig. 5 ▸(*b*). In Fig. 5 ▸(*c*) the intensity distribution along the 220 reciprocal direction is compared for both Bloch wave and multislice simulations. The intensity profile was extracted along the annotated box region shown in Fig. 5 ▸(*a*), and the intensity of the unscattered beam was normalized for a direct visual comparison. The minor quantitative differences between the two profiles are believed to be largely due to numerical rounding errors in the inelastic scattered wavevectors (note that the pixel size of the diffraction pattern is an order of magnitude larger than 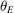). This is also likely to be the reason why the HOLZ ring in the multislice simulation [Fig. 5 ▸(*b*)] has asymmetric intensity.

**Figure 6:**
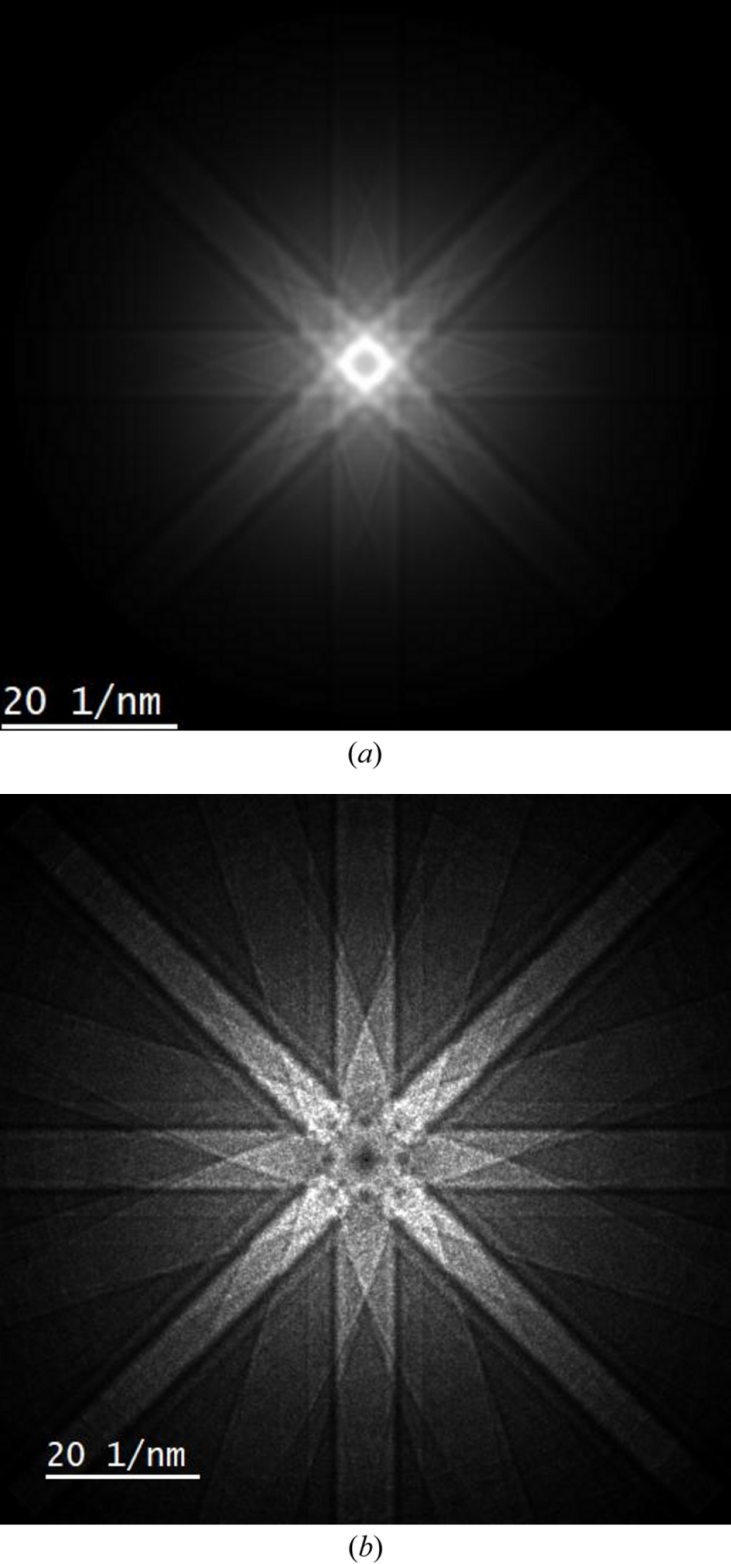
(*a*) Single scatter phonon intensity distribution for a 500 Å-thick Si [001] specimen simulated using the physical optics Bloch wave model. (*b*) shows the phonon diffuse intensity for the same specimen calculated using multislice frozen phonons and the quantum excitation of phonons model.

The single scatter phonon diffuse background, calculated using physical optics Bloch waves [equation (23)], is shown in Fig. 6 ▸(*a*). This is a considerably larger calculation than plasmons and took ∼3 days to run on a 32 GB RAM PC. A total of 147268 scattering vectors were simulated in Fig. 6 ▸(*a*), covering phonon scattering angles up to 100 mrad (Mendis, 2024*b* ▸). In contrast, plasmons required only 140 scattering vectors for a maximum scattering angle of 2 mrad (= 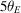). The equivalent multislice simulation was computationally too expensive to perform. This is easily understood by considering the computational cost for each method, *i.e.*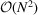 and 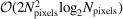 per slice for physical optics Bloch wave and multislice, respectively. For the simulation parameters in this study (Section 3), the multislice simulation time is increased by an order of magnitude.

A more efficient multislice method for simulating TDS is the quantum excitation of phonons model (Forbes *et al.*, 2010 ▸). Fig. 6 ▸(*b*) shows the phonon diffuse intensity calculated using this method. Strictly speaking, Fig. 6 ▸(*b*), which includes multiple phonon scattering, cannot be directly compared with the single scatter distribution in Fig. 6 ▸(*a*). From Poisson statistics, single and higher-order phonon scattering constitutes 6.1% and 0.2% of the total electron intensity for our sample, respectively. Multiple scattering is therefore weak (*i.e.* an order of magnitude smaller), but not entirely negligible. This explains why the diffuse intensity is spread out to higher scattering angles in Fig. 6 ▸(*b*). It may also be the reason why the higher-order 620 Kikuchi bands are visible in Fig. 6 ▸(*b*), while being only weakly present in Fig. 6 ▸(*a*), and revealed only after adjusting the contrast. Nevertheless, it is clear that the gross features in the TDS are correctly reproduced by the physical optics Bloch wave model.

## 5. Summary

Matrix operators for the multislice phase grating and propagator functions are derived from the Bloch wave structure matrix. Dynamical scattering can therefore be simulated using a physical optics approach, where the specimen interaction and free space propagation of the electron beam are decoupled. This is mathematically identical to multislice, although practical limitations in the implementation of each simulation technique lead to small numerical differences. The physical optics Bloch wave method is ideal for many computationally demanding simulations in 4D STEM (imaging modes), 3D ED (precession and rotation electron diffraction), as well as phonon and plasmon inelastic scattering. In all these cases the specimen remains fixed, while only beam propagation within the specimen changes between successive simulations (*e.g.* individual wavevectors within a STEM probe or precession cone). Diagonalization of the Bloch phase grating function can then be re-used, depending on the details of Bragg beam selection.

The computational complexity of the physical optics Bloch wave method scales as 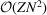, where *Z* is the number of slices and *N* the number of diffracted beams. For ‘thin’ specimens (small *Z*) a physical optics simulation could be more efficient than matrix diagonalization in a standard quantum-mechanical Bloch wave calculation, which scales as 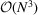. The cut-off thickness is specimen dependent, and increases with the unit-cell dimensions (*i.e.* large *N*). The two formulations of Bloch wave theory, *i.e.* quantum-mechanical and physical optics, provide greater flexibility for a broader class of dynamical diffraction simulations. The quantum-mechanical approach is desirable for small unit cell crystals, while physical optics calculations have better performance for large unit cells. Finally, for perfect crystal specimens, the physical optics Bloch wave method could also at times outperform multislice, since only the important Bragg reflections in the otherwise sparse diffraction plane are calculated.

The computer code for this work is available open access from the Durham University research data repository (doi: 10.15128/r2m613mx59t).

## Supplementary Material

Supporting information. DOI: 10.1107/S2053273325000142/tw5012sup1.pdf
