# Supplementary material for: A physical optics formulation of Bloch waves and its application to 4D STEM, 3D ED and inelastic scattering simulations
Source: Acta Crystallogr A Found Adv. 2025 Jan 30;81(Pt 2):113–23. doi: 10.1107/S2053273325000142 (PMC11873815; doi:10.1107/S2053273325000142)
Supplement: Supplementary file 1 [file a-81-00113-sup1.pdf]

Acta Cryst A
FOUNDATIONS
ADVANCES

Volume 81 (2025)

Supporting information for article:

A physical optics formulation of Bloch waves and its application to 4D STEM, 3D ED and inelastic scattering simulations

Budhika G. Mendis

S1. Higher order term derivation of Bloch phase grating matrix

Here we complete the proof that the Bloch phase grating matrix is equal to a convolution of the Fourier transformed, multislice phase grating function. The general expansion term in the right-hand side of Equation 13 (main text) can be written as:

$$\mathcal{F}\left[\exp\left(i\sigma V_p(\mathbf{R})\right)\right] = \int \left[1 + i\sigma V_p(\mathbf{R}) + \cdots\right] e^{-2\pi i \mathbf{u}\cdot\mathbf{r}} d\mathbf{r}$$

$$= \delta(\mathbf{u}) + i\sigma\Delta z \sum_{\mathbf{h}\neq\mathbf{0}} V_{\mathbf{h}} \delta(\mathbf{h}-\mathbf{u}) + \cdots + \frac{(i\sigma\Delta z)^n}{n!} \sum_{\mathbf{h}_1,\ldots,\mathbf{h}_n\neq\mathbf{0}} V_{\mathbf{h}_1} \ldots V_{\mathbf{h}_n} \delta(\mathbf{h}_1 + \cdots + \mathbf{h}_n - \mathbf{u}) + \cdots$$

… (S1)

The reciprocal vectors $\mathbf{h}_1, \ldots, \mathbf{h}_n$ do not include the null vector $\mathbf{0}$, since the mean inner potential is included in the electron wavenumber within the crystal (Section 2.1). Equation 15 (main text) then becomes:

$$\left\{\mathcal{F}\left[\exp\left(i\sigma V_p(\mathbf{R})\right)\right] \otimes f(\mathbf{u})\right\}_{\mathbf{u}=\mathbf{g}}$$

$$= f(\mathbf{g})$$

$$+ i\sigma\Delta z \sum_{\mathbf{h}\neq\mathbf{g}} V_{\mathbf{g}-\mathbf{h}} f(\mathbf{h}) + \cdots + \frac{(i\sigma\Delta z)^n}{n!} \sum_{\mathbf{h}_1,\ldots,\mathbf{h}_n\neq\mathbf{0}} V_{\mathbf{h}_1} \ldots V_{\mathbf{h}_n} f(\mathbf{g} - \mathbf{h}_1 - \cdots - \mathbf{h}_n) + \cdots$$

… (S2)

Using the transformations:

$$\begin{gathered} \mathbf{h}_1 \to \mathbf{g} - \mathbf{h}_1 \\ \mathbf{h}_2 \to \mathbf{h}_1 - \mathbf{h}_2 \\ \vdots \\ \mathbf{h}_n \to \mathbf{h}_{n-1} - \mathbf{h}_n \end{gathered}$$

… (S3)

The last summation in the right-hand side of Equation S2 can be simplified as:

$$\sum_{\mathbf{h}_1,\ldots,\mathbf{h}_n\neq\mathbf{0}} V_{\mathbf{h}_1} \ldots V_{\mathbf{h}_n} f(\mathbf{g} - \mathbf{h}_1 - \cdots - \mathbf{h}_n) = \sum_{\substack{\mathbf{h}_1,\ldots,\mathbf{h}_n \\ (\mathbf{g}\neq\mathbf{h}_1,\ldots,\mathbf{h}_{n-1}\neq\mathbf{h}_n)}} V_{\mathbf{g}-\mathbf{h}_1} \ldots V_{\mathbf{h}_{n-1}-\mathbf{h}_n} f(\mathbf{h}_n)$$

… (S4)

Compare this expression with the general expansion for Equation 16 (main text):

$$\exp\left(2\pi i\Delta z\mathbf{A}_Q\right)f(\mathbf{u}) = \left[\mathbf{I} + 2\pi i\Delta z\mathbf{A}_Q + \cdots\frac{(2\pi i\Delta z)^n}{n!}\mathbf{A}_Q^n + \cdots\right]\begin{pmatrix}\vdots\\ f(\mathbf{h})\\ \vdots\end{pmatrix} \quad \ldots \text{(S5)}$$

For normal plane wave incidence $V_{\mathbf{g}-\mathbf{h}_1} = \frac{2\pi}{\sigma}\left(\mathbf{A}_Q\right)_{\mathbf{g},\mathbf{h}_1}$, $V_{\mathbf{h}_{n-1}-\mathbf{h}_n} = \frac{2\pi}{\sigma}\left(\mathbf{A}_Q\right)_{\mathbf{h}_{n-1},\mathbf{h}_n}$ etc. From Equation S4 it follows that the nth-order term in Equation S2 is equal to the equivalent term in Equation S5. This completes the proof.

S2. 4D STEM probe wavefunction convergence

Figure S1a is the amplitude of the 15 mrad semi-convergence angle, aberration-free STEM probe wavefunction at the specimen entrance surface, calculated according to Equation 28 (main text). The wavefunction is real and radially symmetric. The equivalent STEM probe amplitude and phase calculated using 13,234 partial plane waves is shown in Figures S1b and S1c, respectively. Subtle deviations from perfect radial symmetry are observed, especially for the phase.

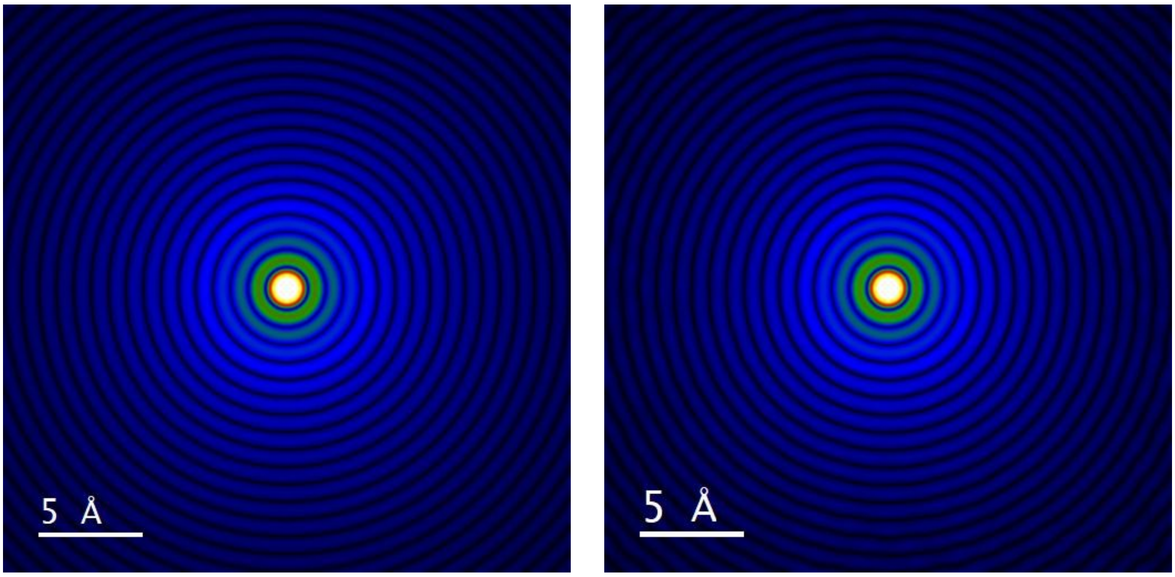

(a) (b)

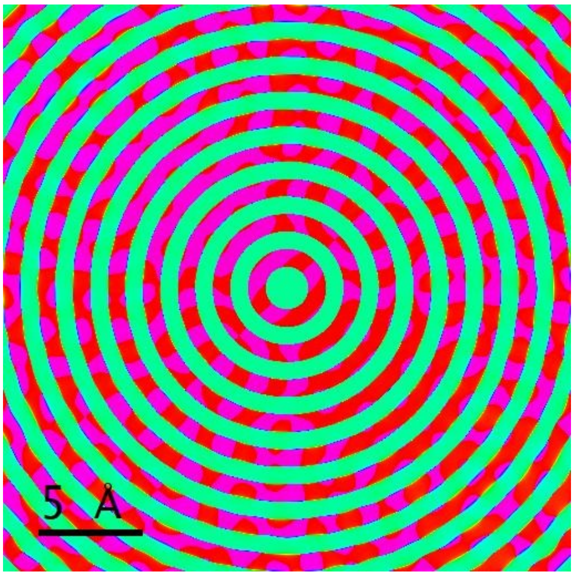

(c)

Figure S1 (a) STEM probe amplitude, calculated using Equation 28 in the main text. Also shown are the partial plane wave simulation results for the (b) amplitude and (c) phase. The amplitude plots in (a) and (b) are displayed on a square root intensity scale to highlight weak features. The colour coding for the phase plot in (c) is green: zero radians, red: -π radians and pink: π radians.

S3. 4D STEM images

Figures 3b to 3d (main text) showed 4D STEM images of Si-[001] calculated using the physical optics Bloch wave method (2 Å slice thickness). Figure below shows equivalent images calculated using the quantum mechanical Bloch wave method. The two sets of images are similar, indicating convergence of the physical optics Bloch wave results.

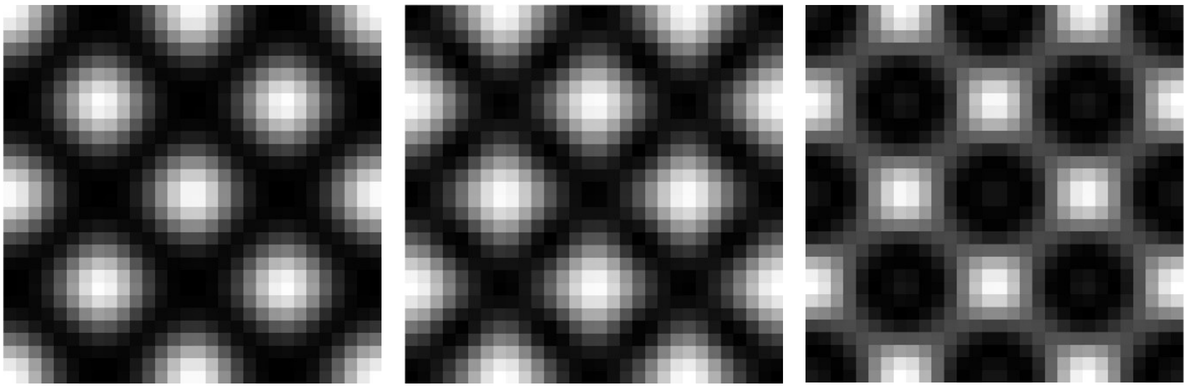

(a) (b) (b)

Figure S2 4D STEM images for (a) bright field (BF), (b) annular bright field (ABF) and (c) medium angle annular dark field (MAADF) imaging modes. The collection angles are 0-5 mrad for BF, 10-15 mrad for ABF and 30-50 mrad for MAADF. The aberration-free STEM probe semi-convergence angle was 15 mrad. The specimen is 500 Å thick silicon, and the field of view spans a single unit cell in [001] projection. The images were simulated using the quantum mechanical Bloch wave method.
